# The Impact of parasitic loss on solar cells with plasmonic nano-textured rear reflectors

**DOI:** 10.1038/s41598-017-12896-1

**Published:** 2017-10-09

**Authors:** Claire E. R. Disney, Supriya Pillai, Martin A. Green

**Affiliations:** Australian Centre for Advanced Photovoltaics, University of New South Wales, Sydney, NSW 2052 Australia

## Abstract

Significant photocurrent enhancement has been demonstrated using plasmonic light-trapping structures comprising nanostructured metallic features at the rear of the cell. These structures have conversely been identified as suffering heightened parasitic absorption into the metal at certain resonant wavelengths severely mitigating benefits of light trapping. In this study, we undertook simulations exploring the relationship between enhanced absorption into the solar cell, and parasitic losses in the metal. These simulations reveal that resonant wavelengths associated with high parasitic losses in the metal could also be associated with high absorption enhancement in the solar cell. We identify mechanisms linking these parasitic losses and absorption enhancements, but found that by ensuring correct design, the light trapping structures will have a positive impact on the overall solar cell performance. Our results clearly show that the large angle scattering provided by the plasmonic nanostructures is the reason for the enhanced absorption observed in the solar cells.

## Introduction

Photovoltaics can meet the global energy demand; with the promise of increased affordability with technology development and economies of scale. To achieve this, cost reductions and increased efficiency are emphasised. Despite advances, photovoltaics have not yet reached their full potential. Silicon (Si) remains the dominant cell material, with cell performance hampered by silicon’s limited light absorption within the wavelength range around its band-edge – especially for thinner layers. Si is also attracting interest as the bottom cell in tandem photovoltaics. Hence there is increased focus on improving light absorption close to the band-gap of Si. Rear light trapping schemes would be crucial for such configurations, considering front textures would impede the growth process of the top cell.

**Figure 1:**
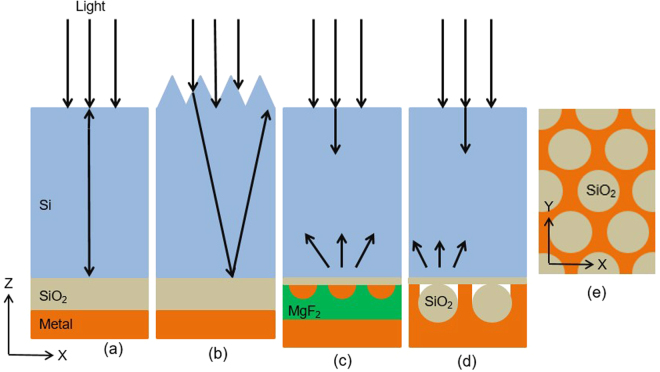
X-Z Cross sections of a cell with (**a**) a standard rear mirror, (**b**) a textured front surface scattering light entering the cell (**c**) rear metal nanoparticles and a rear mirror scattering light back into the cell, and (**d**) metal rear reflector with an array of hexagonally-symmetrical nanospheres. In all cases the rear reflector is separated from the Si by a SiO_2_ spacer layer. (**e**) shows a X-Y view of the nanosphere array at the rear of the cell shown in (**d**).

One method for increasing light conversion within cells is to include structures which ‘trap’ light within the cell, channeling it such that its pathlength through the cell is increased^1^. The rear metal layer used as a back contact for many cells acts as a mirror, giving much of the light increased chance for absorption when it is reflected as seen in Fig. 1(a). Thick Si cells have also used front-surface texturing to modify the angle at which light enters the cell, as in Fig. 1(b), giving it a chance to be ‘trapped’ within the cell - achieving multiple bounces. Besides being unsuitable for tandem cells, such texturing negatively impacts the electrical characteristics of the cell, as increased surface area is associated with increased surface recombination. It is also unsuited for thin solar cells as the feature size of the texturing is similar to the thickness of these cells^2^.

Plasmonic light trapping represents an alternative strategy to more effectively couple light into the cell while avoiding detrimental impacts of texturing, or can be used along with front texturing for thicker cells. Plasmonic light trapping can result from excitation of surface plasmons, leading to greatly enhanced electric fields and scattering of light into a cell’s active layer^3^. Numerous types of plasmonic light trapping structures have shown enhanced performance for solar cells^4–9^. Front-surface structures aim to minimize reflection from a cell’s front surface^10^, while rear-surface structures aim to maximize large-angle back-scattering of light into the cell from its rear surface^11,12^. Rear located structures that don’t increase the surface area of the semiconductor layer are this paper’s focus. Two such structures are shown in Fig. 1(c and d). The former is an array of metal nanoparticles (MNPs) on a cell’s rear surface, coated with magnesium fluoride (MgF_2_) and an additional rear mirror^13^, while the latter involves a continuous metal layer textured using an array of Silica nanospheres (NSs)^14^. These metal films incorporating NSs have previously shown the potential to significantly enhance photocurrent (*J*_*ph*_) in thin solar cells^15^. A key advantage of these NS-textured rear reflectors is that they offer both the light trapping enhancements of the MNPs, with the continuity of a rear mirror. This metal continuity allows them to serve as the rear contact for a solar cell. Such reflectors can be fabricated using cheap spin-coating processes and no significant added processing or material costs, making them viable and interesting. Nanostructured Ag rear electrodes on organic solar cells' active layer have demonstrated significantly improved device performance^16^.

Despite the potential enhancements offered to photocurrent, concerns remain about increased parasitic absorption losses in metal that could occur in these types of plasmonic light trapping structures^17,18^. Even standard metal rear reflectors in solar cells suffer parasitic losses. A thick dielectric layer such as aluminium oxide (Al_2_O_3_)^19^ or SiO_2_ has been used for passivation and to offer an additional interface for reflection into Si, minimizing light interaction with the metal. Studies are focusing on replacing plasmonic structures with Mie scattering structures to overcome the parasitic absorption losses^20^. The aim in light trapping is to maximize absorption in the cell’s active layer, so parasitic absorption in metal would seem to be negative in these designs. Indeed, metal films with arrays of nanovoids resembling those shown in Fig. 1(d) have previously shown potential for uses maximizing coupling of light into the metal itself^21^. However, heightened absorption was localized over narrow-band peak wavelengths of light, determined by the resonances of the void^22^. Altering the void size and spacing alters the resonant wavelengths and their strength^15,22^. These structures can provide both strong absorption into the metal and effective light trapping in the cell, warranting deeper analysis of the balance between these competing characteristics.

The impact of unwanted parasitic losses in the metal must be weighed against potential cell performance enhancements these structures can provide^23^. Some studies tended to consider absorption in the solar cell, while neglecting absorption in the metal. Several investigations concluded parasitic absorption is problematic for MNPs in some cases^24–26^, with low parasitic absorption highlighted as a key benefit of certain designs^27–29^. Some experimental work also found that for MNPs, parasitic losses are insignificant in the visible part of the spectrum, but increase in NIR wavelengths^30^. These losses were attributed to multiple internal reflections of light inside the Si film, due to rapid reduction of the semiconductor’s absorption co-efficient, although useful absorption was also enhanced. Simulations reveal much more about the underlying mechanisms that lead to these results, allowing for deeper understanding to better guide structure optimization.

## Results

### Simulation Method

3D finite-difference time-domain simulations using Lumerical^31^ explored the impact of parasitic absorption in rear metal light trapping structures for solar cells. These simulations reveal the spatial distribution of light absorption into each material, the idealized photocurrent (*J*_*ph*_) of thin Si cells implementing these designs, as well as the angles light is back-scattering into the cell from these rear reflectors. 2 µm thick Si cells were used for the photocurrent calculations to allow for broadband investigation of enhancement not possible in thick cells. Both absorption enhancement and scattering are key to understanding the impact of these structures on cell performance. The main structures simulated were rear mirrors with NSs as shown in Fig. 1(d), owing to their ability to serve as both rear contact and light trapping structure. However MNP arrays with mirrors as shown in Fig. 1(c) were also simulated to compare parasitic absorption in metal nanoparticles or nano-textured metal films. A unit cell with periodic boundary conditions is used to simulate a larger array.

Non-polarised light absorption into silicon (Si) was integrated across the AM1.5 G spectrum in order to calculate the *J*_*ph*_ for each simulated structure as in previous work^15^. For the MNP case, a rear array of hemispherical silver (Ag) MNPs was simulated, coated with a 500 nm thick MgF_2_ layer and rear Ag mirror similar to structures that have demonstrated enhancements^13^. For the NS case, a rear-located hexagonal array of silica (SiO_2_) nanospheres was simulated, coated with a continuous silver film. Line-of-sight application of the metal over these NSs gives their resulting texture of the metal film a ‘half-capsule’ shape^8^ providing a positive impact on the film’s scattering behavior^15^. For both structures, a 20 nm SiO_2_ spacer layer was included at the rear of the Si, selected based on optimization in our previous work^13^.

Optical constants for materials used were taken from existing literature^32^. Optical constant selection for silver required greater scrutiny. Previous simulations used either Palik’s data^32^ or Johnson & Christy’s^33^, but both have limitations when simulating nanostructures, either under or overestimating performance^34^. A new set of optical constants for Ag was published addressing these limitations, allowing more accurate simulation of Ag nanostructures^35^. As such, this was utilized for this paper.

For each structure considered, two types of simulations were conducted. The first isolated interactions with the rear interface of the cell. Each structure was modelled at the rear of semi-infinite non-absorbing Si by modifying its optical constants, allowing analysis of the behavior of each wavelength of light with the rear interface of cell and light trapping structure to be examined. That is, the fraction of light that is absorbed in the metal vs. what is scattered back into the cell can be calculated, as well as the angular profile of this back scattering. This characteristic is independent of cell thickness, and not affected by interference effects that can complicate the behavior of light within thinner cells. However, these simulations do not consider absorption in the Si, and thus a second set of simulations were necessary to examine the impact on *J*_*ph*_.

To observe *J*_*ph*_, simulations modelled the structures at the rear of 2 µm Si, selected as these structures had previously shown strong enhancement for thin cell types^15^. Using thinner Si also allows for exploration of parasitic losses over a wider bandwidth of light, down into the visible part of the spectrum. Absorption in both the Si and Ag was isolated for each structure and integrated across the AM1.5 G spectrum to obtain the *J*_*ph*_. These were compared against the *J*_*ph*_ for the same cell with a standard rear reflector; an Al or Ag mirror with a 100 nm thick spacer layer, providing a fairer assessment of the enhancement offered by these structures against standard reflectors.

**Figure 2:**
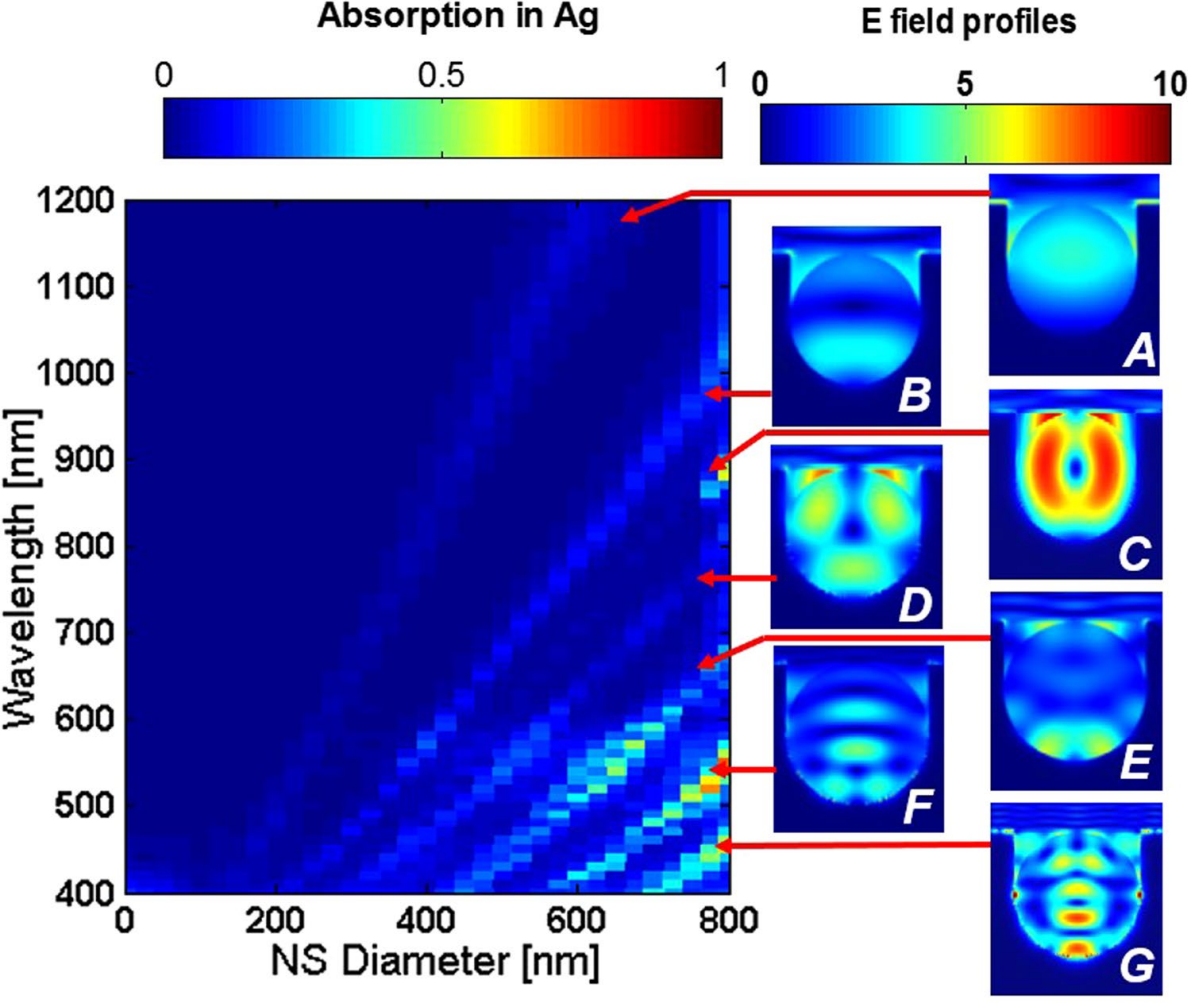
Absorption into an Ag reflector at the rear of semi-infinite non-absorbing Si, with embedded arrays of NSs of varying sizes and an 800 nm period. Absorption peaks emerge at resonant wavelengths. As the NS size increases, these resonances strengthen and red-shift. E-field profiles for each resonance are shown in the insets.

Results of the rear interface simulations give the fraction of light reaching the rear interface which will be lost in metal, for each structure at each wavelength. This case is equivalent to no light being absorbed in the silicon prior to reaching the rear interface, allowing us to examine the resonant behavior of these plasmonic structures at shorter wavelengths well absorbed by even thin layers of Si, unlikely to ever reach the rear interface of a cell. A selection of results for the NS structures is shown in Fig. 2. In this case, an 800 nm NS array period was chosen. This was identified as a relatively high performing configuration in previous simulations^15^. The minimum diameter of the NSs was ‘0 nm’, equivalent to the base case of a rear mirror and thin spacer layer. While the maximum diameter was 800 nm i.e. equal NS size and period, leaving the NSs densely packed and touching. The mirror-only case gives slightly shifted interference peaks in absorption depending on whether the SiO_2_ spacer layer is 20 nm thick (‘0 nm’ case) or 100 nm thick reference as in traditional cells. However, these result in the same simulated *J*_*ph*_. Thus these can be considered equivalent for comparing *J*_*ph*_, but the interference peak shifts must be taken into consideration when examining the absorption at a specific wavelength.

Several resonances (*A-E*) were identified for these structures, with *A* representing the lowest-order mode and each letter representing progressively higher order modes. These modes red-shift and strengthen with increasing NS diameter. Larger NS sizes > 1 µm with correspondingly larger periodicity cause higher-order modes to shift into the NIR wavelength range of interest as the red-shifting trend continues with NS size increase as shown in Fig. 2. However, the greatest advantage seems to occur when lower-order modes occur in the NIR wavelength range, associated with greater scattering back into the Si. A mirror alone evidently results in the lowest parasitic losses in Ag. Thus, this would be the preferred reflector if only parasitic absorption losses are considered. As NS size increases, so do parasitic losses in metal, becoming more broadband. If the aim is only to minimize parasitic absorption, then selection of NS size with no resonances in the wavelength range of interest for a given thickness cell – such as the 200 nm NS case in Fig. 2 - would be necessary.

**Figure 3:**
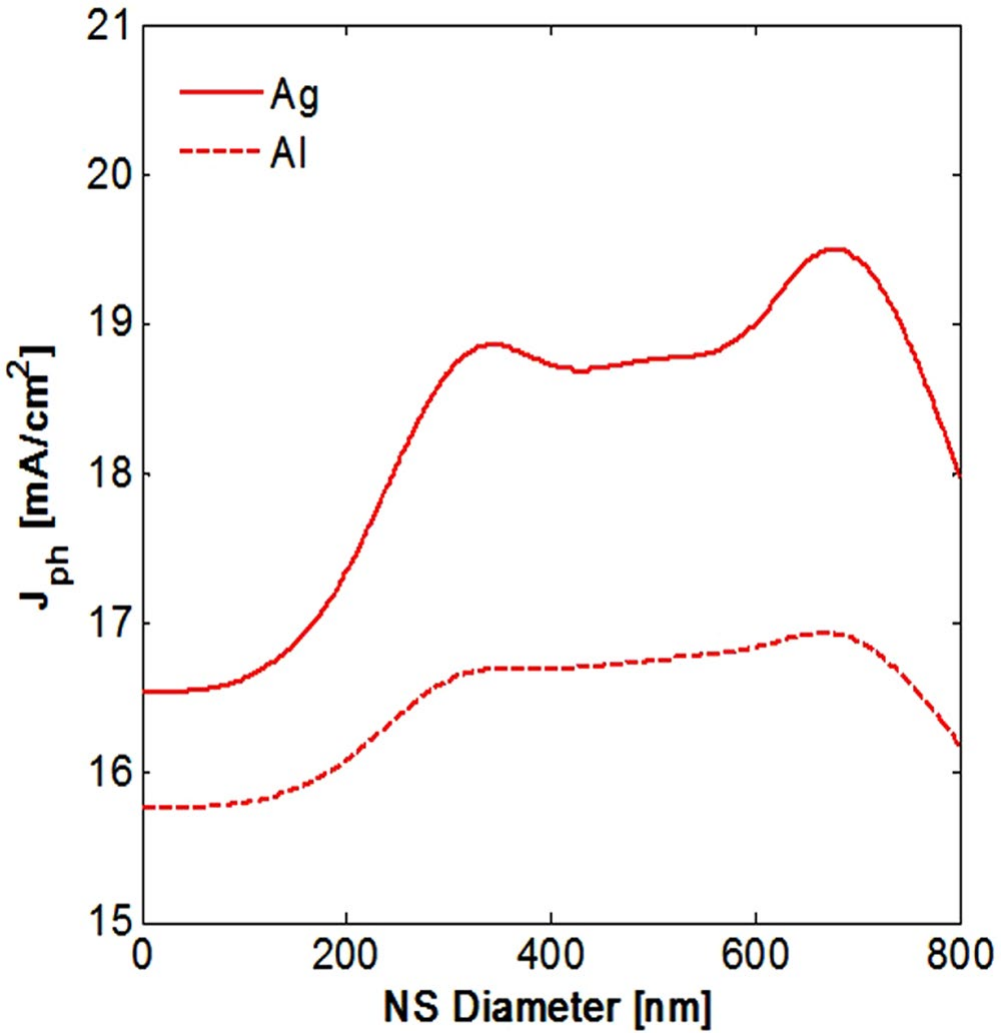
Photocurrent for 2 µm thick Si with rear Al or Ag with embedded silica nanospheres of varying sizes with 800 nm array period. Despite higher absorption losses in the metal, *J*_*ph*_ is maximized with larger NSs. Al reflectors give significantly reduced enhancements to *J*_*ph*_.

Simulations on 2 µm thick Si determined the impact of these NSs on photocurrent. These compare the impact of the large parasitic losses in metal with the enhancement offered by these structures over the widest wavelength range possible. Selecting a thin cell allows the broadest wavelength range of light to reach the cell’s rear surface. These simulations used the same size variations shown in Fig. 2, giving the *J*_*ph*_ for different NS sizes shown in Fig. 3. Once again, the ‘0 nm’ case has the same *J*_*ph*_ as a rear mirror alone, providing a baseline for comparison.

Aluminium was also considered for the rear reflector. Previous work identified that this would provide significantly poorer enhancements to *J*_*ph*_ relative to an Ag based structure^15^, and understanding whether parasitic losses contribute to this difference is of interest. Figure 3 shows that trends in *J*_*ph*_ enhancement for different NS sizes were similar with either metal. As expected Al offered lower enhancements. The patterns of highly absorbing resonances in the metal from Fig. 2 were comparable for Al case (not shown here). Al structures inherently suffered significantly greater absorption losses in the metal even for the mirror case. These underlying parasitic losses for Al structures seem likely accountable for large differences in enhancement offered by the two metals.

**Figure 4:**
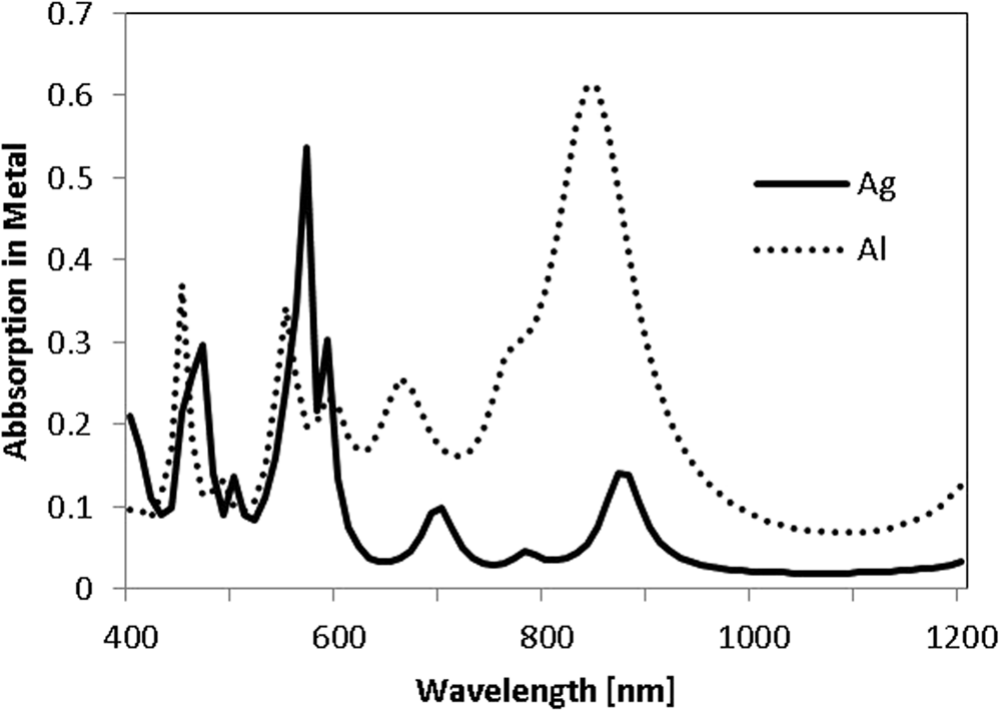
Absorption into Ag or Al reflector at the rear of semi-infinite non-absorbing Si, with embedded arrays of 675 nm NSs and an 800 nm period. Al has a significantly increased base absorption into the metal.

Selecting the best NS size from Fig. 3 - 675 nm NSs with an 800 nm period - we can then compare losses in the metal for the non-absorbing Si case with either Ag or Al as shown in Fig. 4. This reinforces that there is dramatically higher absorption in Al than the Ag, especially in the NIR range. These underlying absorption losses in Al limit their potential as rear reflectors. However, they may be suitable for thick cells, where only larger wavelengths are relevant.

Contrary to expectations, the simulations on 2 µm Si reveal that geometries with the lowest parasitic losses in the metal provide the poorest *J*_*ph*_ enhancement. While all geometries of the NSs have a positive impact on cell performance, the greatest enhancement in Si is caused by 675 nm NSs with an 800 nm period for Ag. These features also showed significantly increased parasitic losses in Ag relative to the smaller NS cases or mirror. Strong enhancements persist despite raised parasitic absorption, and only when the NS size becomes very close to the array period does increased parasitic absorption negate the potential enhancements offered by these structures. This is due to highly absorbing sharp features in the metal. An NS diameter of around 100–200 nm smaller than the array period offers the best results. A more thorough exploration of the impact of size and period variations was previously conducted^15^.

A potential explanation for this relationship could be that despite suppressed scattering back into Si at resonance, this is outweighed by strong scattering enhancement at wavelengths between resonances. To investigate whether this is the case, we quantified the absorption into Si for each wavelength. If this hypothesis were true, then there would be suppressed absorption in Si at resonances *A-E*, but heightened absorption at wavelengths between them. Though we did find significantly heightened absorption into Si at wavelengths between resonances, strong absorption enhancement in Si was also found at the resonant wavelengths themselves. A selection of these results is shown in Table 1, where absorption into Si for selected wavelengths is shown for the mirror case, with 200 nm NSs or 675 nm NSs. The wavelengths selected were at resonances *B, C, D*, or *E* for 675 nm NSs. Additionally, a wavelength midway between resonance *A* and *B* was included, as resonance *A* lies beyond the wavelength range of interest for this NS size. These were compared against 200 nm NSs as these wavelengths were not near resonances for that size, and against the Ag mirror which provides the baseline. Since specific wavelengths are considered, using the mirror with 100 nm thick spacer layer would provide distorted results due to the shifted interference bands, so the 20 nm case was used to give a direct comparison.

**Table 1:** Absorption in Si and Ag at various wavelengths, for 2 µm thick Si with either a rear 20 nm spacer layer and Ag mirror, or including 200 nm or 675 nm diameter nanospheres (800 nm period) in the Ag.

Wavelength [nm]	Absorption in Si (%)			Absorption in Ag (%)		
	Mirror only	With 200 nm Nanospheres	With 675 nm Nanospheres	Mirror only	With 200 nm Nanospheres	With 600 nm Nanospheres
565	52.3	52.6	52.7 *(E)*	0.1	0.1	1.5
700	24.1	25.6	44 *(D)*	0.2	0.3	4.5
780	41.1	50.1	56.5 *(C)*	0.9	1.2	3.1
875	5.9	7.7	9.2 *(B)*	0.3	0.6	7.8
1050	1.3	1.7	20.2 *(B→A)*	0.5	0.7	4.6

Despite increased absorption losses in the Ag, the 675 nm NSs provide a much greater enhancement to absorption in Si. These wavelengths were selected as they correspond to peaks in absorption for the 675 nm case, but not for the 200 nm case as well as the midpoint between resonances *B* → *A*. Each absorption in Si is labelled with the resonance it corresponds to.

The results in the table reveal that for each case, there was significantly increased enhancement to the percentage of light absorbed in Si at resonance. This is especially true for those resonances falling in the NIR wavelength range. Where the resonance falls below 600 nm, the difference in absorption is much less marked, as more light is absorbed prior to reaching the cell’s rear surface. In addition to higher absorption in Si, as expected, the 675 nm NSs have significantly increased parasitic losses in Ag at these resonances. As such, both higher absorption into the metal and enhanced absorption into the Si result at these resonances. Similar trends were observed when aluminum was simulated instead of silver. This suggests these trends are common to nanotextured metals with these geometries, rather than specific to Ag. It is evident that the increased absorption in Ag comes not at the expense of absorption in Si, but rather from the portion of light that would otherwise have been lost out the front of the cell due to specular reflection from the planar metal surface.

**Figure 5:**
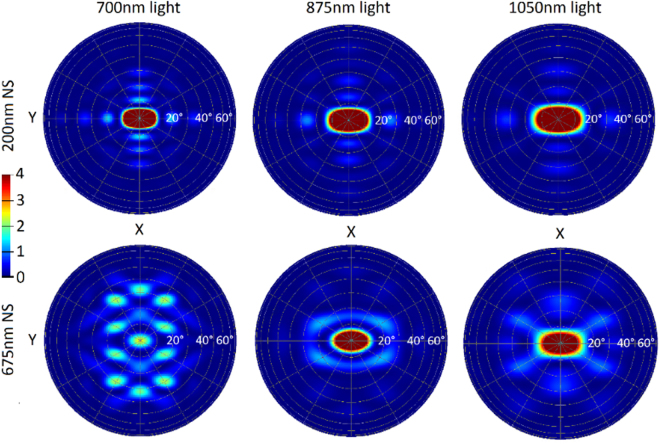
Far-field scattering plots showing the angles at which light is scattered back into the Si from the rear interface of the cell. The rear reflector used included 200 nm or 675 nm diameter NSs embedded in a silver film at the rear of semi-infinite Si. The array period of the NSs was 800 nm. The wavelengths selected were at resonance for the 675 nm NS case, and off resonance for the 200 nm NS case. 700 nm resonance *C*, 875 nm resonance *B*, 1050 nm light - between resonance *A* and *B -* shows the large boost in large angle scattering between resonances.

With these results, we can discount the previous hypothesis, and investigate what allows these structures to provide simultaneous enhanced coupling of light into Si, and increased losses in the metal. Returning to the first set of simulations on non-absorbing Si, it is possible to determine the scattering angles of light from the cell’s rear interface. Far field projections of the light scattered can be plotted for each wavelength and for each structure type. Figure 5 shows far field plots of the light scattered from the rear interface, for several of the wavelengths previously selected for Table 1. The wavelengths for resonances *D, B*, and the midpoint between resonances *A* and *B* are shown for an array of 675 nm NSs. Once again, these are both compared to the 200 nm NS case, for which these wavelengths are off-resonance. It is unnecessary to include the plots for the mirror case, as all light is returned normally in that case and none scattered at larger angles.

These results reveal that at resonance, despite a reduced fraction of light back-scattered into Si, the fraction back-scattered at large angles is greatly increased. This large-angle scattering persists broadly surrounding these resonances and between them, acting as a counterbalance to increased parasitic losses, allowing the enhanced absorption in Si to be maintained across a wide range of wavelengths. This large-angle scattering increases the chance of light achieving multiple bounces and being absorbed in Si rather than escaping out of the front of the cell. The 200 nm NS case results in a greater fraction of light being scattered at smaller angles, and thus cannot benefit from the same degree of pathlength enhancement offered by the larger NSs. For the case where NS size equals the array period, the large-angle scattering is no longer enough to counterbalance parasitic absorption losses; hence the absorption in Ag becomes too dominant.

**Figure 6:**
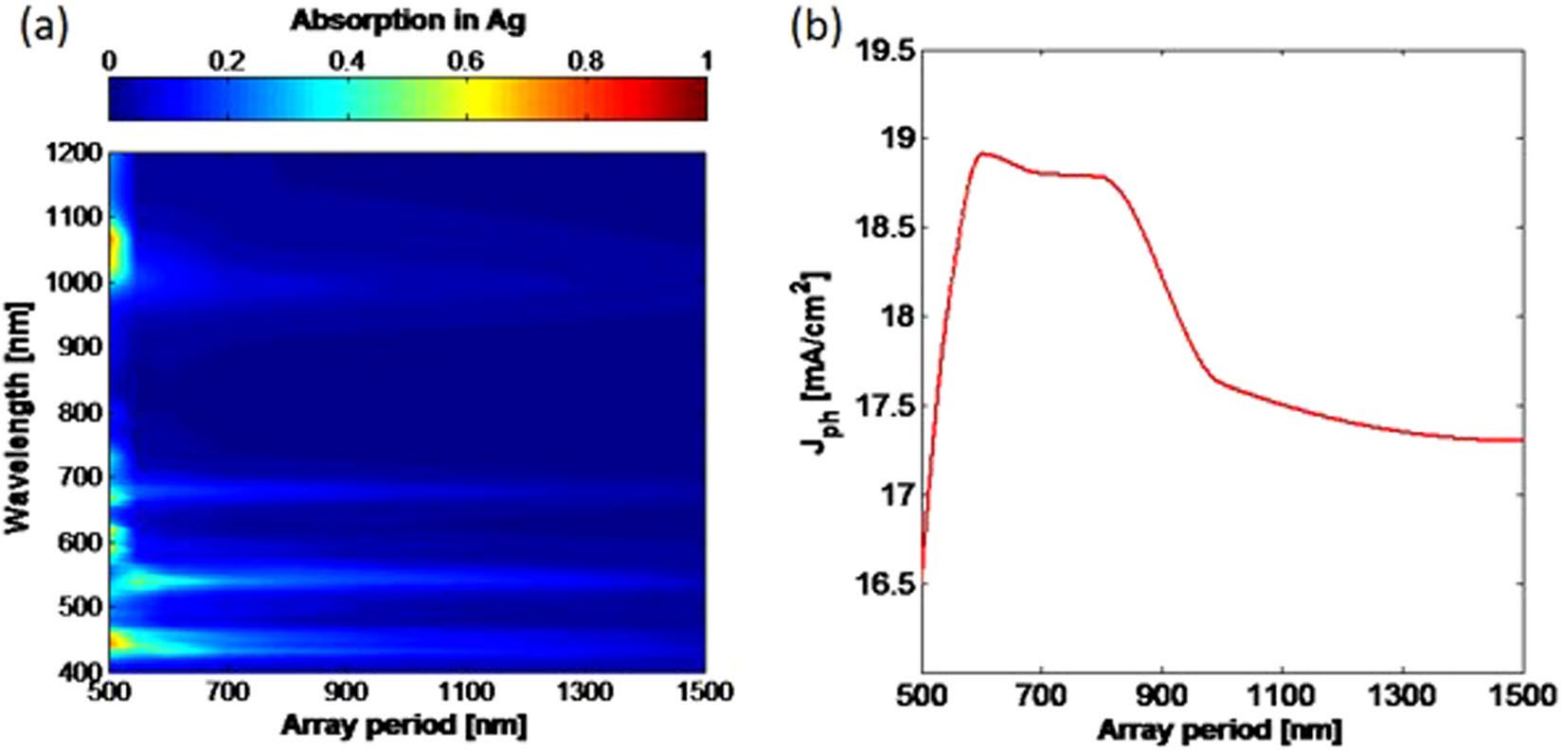
Absorption into Ag with 500 nm nanospheres and varying array period sizes at the rear of semi-infinite non-absorbing Si. As the period increases, the wavelengths at which the absorption peaks occur are maintained, but their magnitude is reduced. (**b**) *J*_*ph*_ for 2 µm thick Si for the case of 500 nm nanospheres with varying array period.

This trend is maintained across varying array periods. Similarly, in addition to understanding the influence of NS size on scattering behavior, it is prudent to investigate the impact of varying array period while keeping NS diameter fixed. Figure 6(a) shows the absorption in Ag at each wavelength for 500 nm NSs with array periods between 500–1500 nm, but similar trends are seen for any size of NS considered. As the array period increases, the resonant wavelengths do not shift. However, as the period increases, the strength of these resonances decreases gradually. As can be seen in Fig. 6(b), there is a relatively broad peak in *J*_*ph*_ for periods between 600–850 nm. Above this, *J*_*ph*_ decreases more slowly, giving an enhancement of ~1 mA/cm^2^ relative to a rear mirror. So, despite significant absorption losses in Ag for periods 100–200 nm larger than the NS diameter, they significantly outperform larger periods. Figure 6(b) shows *J*_*ph*_ for the cases where array size and period are equal – the poorest performing case – is >16.5 mA/cm^2^. This is similar to the *J*_*ph*_ of a cell with a rear mirror alone (~16.5 mA/cm^2^), showing that even the worst performing NS case does not lead to reduced photocurrent.

As the NS array period increases, the angles at which light is scattered are narrowed, but the overall amount of light scattered back into the cell is increased. Once again this leads to a trade-off, whereby the aim is to balance the amount of scattering against the desire for greater large angle scattering. The interplay between these two factors varies depending on the NS size selected, and so some NS diameters will have broader or sharper ranges of optimal periodicities^15^. In this case, the larger periods still provide reasonable scattering above the critical angle for Si, and this is why they are still able to provide significant enhancement to *J*_*ph*_ relative to the mirror case.

**Figure 7:**
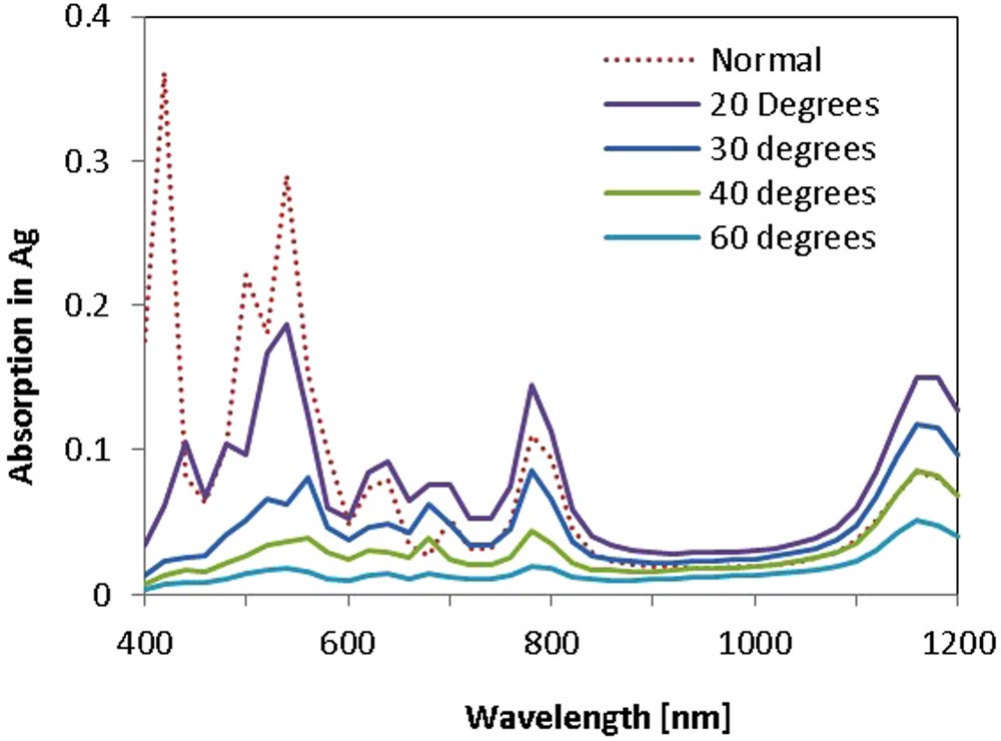
Absorption into Ag for 600 nm NSs with a 700 nm period located at the rear of semi-infinite non-absorbing Si, for varying angles of incoming light. Though the location of resonances does not change, their magnitude decreases as the incoming angle of light deviates further from normal.

**Figure 8:**
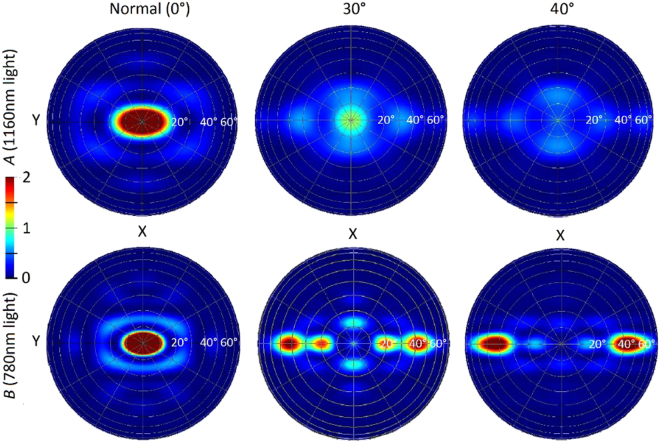
Far-field scattering plots showing the angles at which light is scattered back into the Si from the rear interface of the cell, for different incoming angles of light. The rear reflector used included 600 nm NSs embedded in an Ag film at the rear of semi-infinite Si. The array period of the NSs was 700 nm. The wavelengths selected were at resonances (**A**) and (**B**). Although the scattering patterns vary as the incoming angle of light does, significant large angle scattering is maintained across all cases.

Another aspect to consider is how these structures will respond to light incident from angles other than standard normally-incident light. This would be especially relevant for thicker cells with front-texturing. Previous work suggested a low sensitivity to the incoming angle of light for these structures^15^, showing they could provide *J*_*ph*_ enhancement for thin cells regardless of the light’s incoming angle. However, looking at the rear surface only allows greater insights into how the resonances are impacted by this variation in incoming angle, and to study the impacts independent of cell thickness. Figure 7 compares absorption into Ag for different angles of incoming light, taken from these rear-surface-only simulations. Changing the incoming angle of light does not greatly impact the wavelengths at which resonances occur. However, the magnitude of the resonances diminishes as the incoming angle of light deviates further from normal. This is particularly true of those resonances occurring at shorter wavelengths, with a smaller change evident in resonance *A*, which occurs in the NIR wavelength range. Figure 8 shows the far-field profiles at resonance *A* and *B* for three incoming angles of light. The behavior of the scattering changes as the incoming angle of light shifts, but that the desired large angle scattering remains significant, regardless of the incoming angle of the light. This supports the hypothesis that these resonances are a set characteristic of the geometry, and are not very sensitive to the incoming angle of light. This further supports existing evidence that these structures should offer enhancement to *J*_*ph*_ regardless of incoming angle of light.

**Figure 9:**
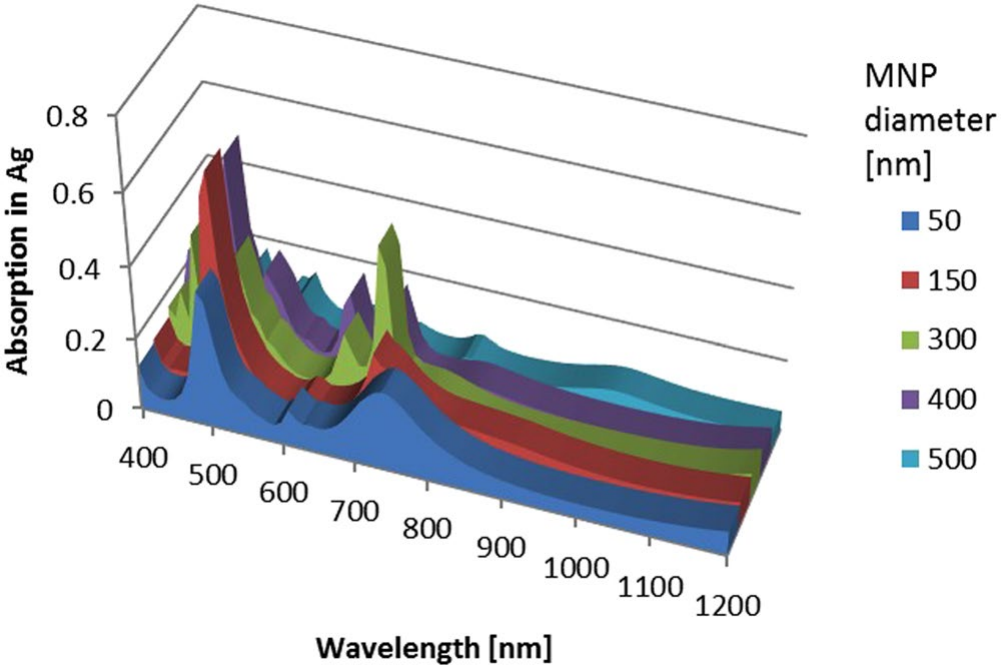
Absorption into Ag MNPs and rear mirror for varying MNP sizes with an array period of 500 nm, located at the rear of semi-infinite non-absorbing Si.

Although these simulations elucidate the behavior of metal films with nanocavities, there are many other types of plasmonic light trapping structure, the more popular being metal nanoparticles. Simulations were also undertaken to understand the balance between parasitic absorption and scattering for rear arrays of hemispherical MNPs coated with MgF_2_ along with a rear mirror. Some experimental work looking at parasitic absorption in MNPs had previously been conducted^30^, finding substantially increased parasitic losses in the metal for the NIR range, but also strong absorption enhancement in the Si giving a strong overall performance enhancement. It is well known that scattering and absorption co-exist in metal nanoparticles, however one may dominate the other which is determined by the feature size^36^. In smaller nanoparticles (~50 nm and less) absorption dominates whereas for larger nanoparticles (>~50 nm) scattering dominates. As size increases the scattering process increases significantly due to its dependence on *r* ^6^ (where *r* is the radius of the nanoparticle. In this study A period of 500 nm was selected for the square arrays of MNPs, and MNP diameters from 50 nm to 500 nm were simulated, relatively similar to previously identified high performing MNP arrays^37^. The patterns of resonances were more complicated in this case, as shown in Fig. 9. Showing the absorption into the Ag MNPs and mirror at the rear of semi-infinite non-absorbing Si. For most sizes there was a strongly absorbing resonance at around 700 nm light. 50 nm MNPs had a negative impact as compared to a rear mirror, which is to be expected due to the absorption dominating for MNPs of this size. For larger MNPs the 300 nm NPs offered the best performance with a *J*_*ph*_ of 19.48 mA/cm^2^. The results for 500 nm and 400 nm MNPs was also fairly close to this at 19.26 mA/cm^2^ and 19.04 mA/cm^2^ respectively.

**Figure 10:**
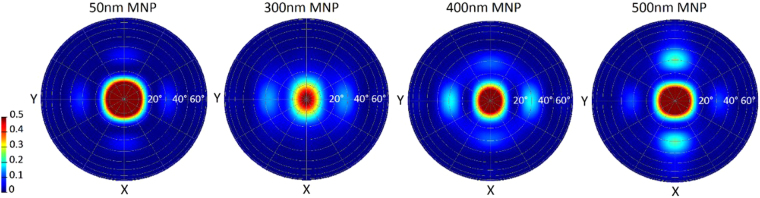
Far-field scattering plots showing the angles at which 700 nm light is scattered back into the Si from the rear interface of the cell for varying sized hemispherical Ag MNPs coated with 500 nm of MgF_2_ as well as a rear mirror. The 300 nm and 400 nm MNPs have an absorption peak in Ag at this wavelength while the other sizes do not.

Once again, we can examine the absorption at resonant wavelengths to examine whether the same relationship between large angle scattering and absorption in metal occurs with these structures as compared to the NSs. Figure 10 shows the far field scattering plots for 700 nm light for several different MNP sizes. The 300 nm and 400 nm MNPs have a resonant peak in absorption in Ag at this wavelength, while the 50 nm and 500 nm MNPs are not. The interpretation must account for the interplay between the NP size and the interference effects from the rear mirror. The 300 nm, 400 nm, and 500 nm MNPs all still seem to have significant large angle scattering. The absorption into 2 µm Si for this wavelength can be extracted to give further insight. 400 nm MNPs have the highest absorption into Si for 700 nm light, at 34.1%. 300 nm MNPs have a slightly lower value of 31.3%, while 500 nm MNPs have 28.1%. 50 nm NPs which are off-resonance give only 23.1%. The increase in absorption in Si with MNP arrays and mirrors is still clearly evident and well supported by numerous experimental results in literature. These structures seem to be slightly more vulnerable to the negative impact of increased absorption losses outweighing the enhanced scattering offered due to having smaller features which act as absorption hotspots. Random MNP arrays, where size variation leads to small or sharp features, can similarly become sights of extremely elevated absorption. This is like the sharp features seen in the metal rear reflectors with NS size equal to array period. This was found to also be the case for cylindrical SiO_2_ tubes in metal films, where closely packed tubes with sharp defined features caused high levels of absorption in the metal film^38^ Similarly the excitation of surface plasmon polaritons at a roughened Ag surface with sharp features was found to lead to significant increases in absorption in the metal^39^. Thus careful consideration would be needed to ensure that the features are so chosen that resulting light trapping structure would offer a benefit rather than being detrimental.

## Conclusion

3D Simulations investigated the impact of parasitic losses in the metal of several plasmonic light trapping structures for solar cells following concerns that the heightened parasitic absorption losses could render them unsuitable for light trapping in solar cells. Simulations examined both the fraction of light absorbed into Ag or scattered back into the cell, and the angle of scattering. These results suggest that for nano-textured metal layers, resonances that cause increased losses in the metal are also associated with significantly increased large-angle back-scattering, maximizing the pathlength of light and thus enhancing photocurrent in the cell. Resonant peaks for these types of structures were identified, and their scattering behavior was examined. It was found that for these type of structures, this large angle scattering dominates in most cases, leaving a net positive impact relative to a rear mirror. However this balance between parasitic absorption and large angle scattering should be factored into selecting the correct geometries for nanotextured rear reflectors. For metal nanoparticle arrays coupled with rear mirrors, the balance between these characteristics needs to be considered more carefully. Overall, it can be concluded that higher absorption in the metal need not always be problematic for plasmonic light trapping structures, and may not need to be avoided. Rather, it can potentially be associated with strong enhancements to absorption in the active layer of the cell.

The datasets generated during and/or analysed during the current study are available from the corresponding author on reasonable request.
